# Injury Incidence Before and After the Introduction of Body Checking in Elite Women’s Ice Hockey: A Six-Season Nationwide Insurance-Based Study

**DOI:** 10.1186/s40798-026-01063-4

**Published:** 2026-07-04

**Authors:** Amanda Lahti, Emelie Stenman, Anton Grundberg, Kristina Sundquist

**Affiliations:** 1 Center for Primary Health Care Research, Department of Clinical Sciences, Clinical Research Centre, Lund University, Box 50332, 202 13 Malmö, Sweden; 2 University Clinic Primary Care, Skåne University Hospital, Malmö, Sweden

## Abstract

**Background:**

In 2022, the Swedish Women’s Elite Ice Hockey League (SDHL) became the first women’s league to introduce bodychecking. Using insurance data, this study examined injury incidence before and after the implementation of this rule.

**Method:**

Since 2019, the SDHL has comprised 10 teams with 20–25 players on each. All players in SDHL have license insurance to take care of ice hockey injuries. All injuries that lead to contact with the insurance company are registered in a database. The insurance covers accidental injuries occurring during matches, organized team practices, hockey school sessions, and direct travel to and from these activities. Injury data from all seasons between 2019–2020 and 2024–2025 were analyzed. Injury rates (IR) per 1,000 player-game hours were calculated and compared across seasons and between pre-implementation (2019–2022) and post-implementation (2022–2025) periods.

**Results:**

A total of 120 injuries were recorded among 92 players. IR per 1,000 player-game hours increased from 6.6 (95% CI 3.8–10.7) in season 2021–2022 to 16.7 (11.6–23.2) in 2022–2023, with moderately elevated rates remaining in subsequent seasons. When grouped by period before and after body checking implementation, IR increased from 6.0 (4.4–8.1) pre-implementation to 11.0 (8.6–13.7) post-implementation (*p* < 0.05). The injury incidence was highest during the first season with body checking and declined in subsequent seasons compared with this initial post-implementation peak.

**Conclusions:**

The introduction of body checking in the SDHL was associated with a significant increase in injuries recorded through the insurance system, indicating that this rule change may lead to a higher injury burden. More research on this topic is needed if body checking is to be widely incorporated into women’s ice hockey.

## Background

Women's ice hockey has experienced significant growth in the number of participants during recent decades [1]. A major milestone occurred during the season 2022–2023, when the Swedish Women’s Elite Ice Hockey League (SDHL) became the first women’s league in the world to permit body checking. Body checking has previously been restricted to men’s ice hockey, with permitted age limits typically ranging from 13 to 15 years, depending on the country and region. Although most SDHL players support the introduction of body checking [2] and potential benefits of aligning women’s ice hockey more closely with the men’s game have been proposed [3], this rule change also raises important concerns regarding its association with injuries.

Compared to other team sports, ice hockey experiences high injury rates (IR) [4, 5]. When considering the differences in checking rules, it is not surprising that overall IRs are lower in women’s games than in men’s games [6]. However, although body checking historically has been prohibited in women’s ice hockey, female players experience higher rates of concussions [7]. At the same time, injury patterns in ice hockey are heterogeneous and include a wide range of injury types such as shoulder, knee, and facial injuries. In contrast to men’s ice hockey, where players typically wear visors at a senior level, full-face protection is mandatory in women’s ice hockey, which may protect against injuries such as dental, eye, and facial wound injuries [6].

Research from men’s and youth ice hockey has historically linked body checking to an increased risk of concussion. For example, disallowing body checking among 13–14 year-old players in Alberta, Canada, was associated with a 55% reduction in overall IR [8]. This makes it reasonable to hypothesize that introducing body checking in women’s ice hockey may lead to a different injury panorama than before its introduction.

Despite extensive research on body checking in men’s and youth ice hockey, there is a lack of data on injury incidence following the introduction of body checking in elite women’s ice hockey. Insurance-based injury registries provide a unique opportunity to capture medically treated injuries (in this case, disability following acute injuries requiring medical treatment, and acute injuries necessitating physiotherapy or dental care) across an entire league, independent of team medical staff or self-reported time-loss definitions.

In Sweden, insurance registries have long been used for injury surveillance. All Swedish ice hockey players are insured by the same company (Gjensidige Insurance) [9], which registers all injuries that result in contact with the insurance provider. The insurance covers accidental injuries occurring during games, organized team practices, hockey school sessions, and direct travel to and from these activities.

Within this insurance registry, these injuries have been continuously recorded from 2019 to 2025. The introduction of body checking in the 2022–2023 season created a natural experiment within a stable league structure, allowing longitudinal comparisons before and after the introduction and within the same competitive context. Against this background, the aim of the present study, using nationwide insurance registry data, was to describe time trends in injuries among female elite ice hockey players and to compare injury incidence before and after the introduction of body checking.

## Method

This cohort study is based on insurance registry data from the Swedish insurance company Gjensidige Insurance. All elite female ice hockey players in the SDHL have, since 2019, been covered by a mandatory base insurance provided through Gjensidige, the principal insurance partner of the Swedish Ice Hockey Association and a long-standing main partner of Swedish ice hockey. This licensing insurance is automatically included for all registered players, including those of SDHL. In the season 2022–2023, body checking was introduced in the league.

Body checking rules in women’s ice hockey differ from those applied in men’s ice hockey. According to the International Ice Hockey Federation (IIHF), intentional body checking has traditionally been prohibited in women’s hockey. However, players are permitted to engage in limited puck-oriented contact, such as pushing or leaning against an opponent while competing for puck possession. Rule 101.1 (“Illegal Hit in Women’s Hockey”) specifies that body contact is only allowed when there is a clear intention to play the puck. It explicitly prohibits actions such as stepping or gliding into an opponent to initiate body contact, as well as using the boards to push, pin, or otherwise eliminate an opponent from play [10].

In the 2022–2023 season, the Swedish Women’s Elite Ice Hockey League (SDHL) introduced body checking within this regulatory framework. In practice, this allowed more explicit open-ice contact aimed at separating an opponent from the puck, provided that the contact was not delivered from the opposite direction and did not violate safety principles outlined in Rule 101.1.

Thus, while the formal rule structure is based on IIHF regulations, the Swedish implementation represents a shift toward allowing more active body contact situations, particularly in open ice, while still maintaining specific restrictions regarding dangerous play and board contact.

### Study Population

The study includes all licensed female elite players in the SDHL between 2019 and 2025. No exclusion criteria were applied. The league consists of ten teams with approximately 25 players per team. The teams in the league have been largely consistent throughout the study period, with only occasional changes due to relegation and promotion based on sporting results; the only notable exception occurred in the 2022–2023 season when one team withdrew from the SDHL mid-season due to financial difficulties and challenges meeting player requirements, which also affected the total number of matches during this season. The temporary reduction in the number of matches during the 2022–2023 season due to team withdrawal was accounted for in the exposure calculations. In addition, because players may transfer between teams or to other international leagues, the composition of each team and the league also vary over time.

### Calculating Injury Rates (IR)

The IR was estimated by calculating the number of injuries per 1000 h of game participation. Exposure was calculated based on player-game hours.

The insurance registry did not allow the separation of injuries sustained during games and those sustained during team practices. Consequently, all recorded injuries were included, whereas the exposure was based only on game hours (not training hours). Reliable data on training exposure were not available in Swedish registers, but the use of game exposure as a denominator has previously been applied in ice hockey epidemiology [11]. Moreover, capturing total exposure time accurately in team sports presents methodological challenges [12]. In addition, in elite ice hockey, previous epidemiological studies consistently demonstrate substantially higher injury incidence during games compared with training sessions. Therefore, it is reasonable to assume that most insurance-registered injuries occurred during games.

Because the same registration procedures and insurance conditions applied throughout the entire study period, this methodological constraint is unlikely to differentially bias comparisons between pre- and post-implementation periods. Nevertheless, absolute IR that are based only on game hours should be interpreted with caution. Insurance-based injury registries have also been used in previous studies to examine injuries among athletes, providing an alternative to team-based or self-reported surveillance systems [13].

As in previous studies, we defined participation as the time when six players per team were on the ice for a 1-h game (20 min for three periods, ignoring penalty minutes and overtime minutes): 6 players × 2 teams × 1 h × number of games [14–17].

During the 2019–2020 season, 195 game hours were played, with 198, 203, 175, 204, and 207 h recorded in the subsequent seasons 2020–2021, 2021–2022, 2022–2023, 2023–2024, and 2024–2025, respectively. For each season, we used the dates from first to last match played to define the seasons.

### Data Collection

Injury claims were registered continuously from the 2019–2020 season and onward for all injuries that led to contact with the insurance company. Representatives from Gjensidige Insurance recorded and classified all injuries according to standard internal procedures. The Swedish Ice Hockey Federation assisted in identifying players registered in the SDHL during the seasons 2019–2020 through 2024–2025 to enable the insurance company to define the study cohort, where data on the individuals were transferred pseudonymized to the research group. Gjensidige performed only the initial data processing and pseudonymization and was not involved in any statistical analyses for the present study.

### Definition of an Injury

The Gjensidige insurance covers accidental injuries occurring during matches, organized team practices, hockey school sessions, and direct travel to and from these activities, including international participation for up to 45 consecutive days. Only injuries that were covered by the Gjensidige insurance were included in the present study. Pre-existing injuries that occurred before getting the insurance were not covered and therefore not included in this study. If a player sustained multiple injuries, each injury was recorded as a separate injury. The insurance company covered incurred injuries and provided compensation for permanent disability following acute injuries requiring medical treatment, acute injuries necessitating physiotherapy, or dental care in cases of dental trauma. Injuries managed solely within team medical settings without generating an insurance claim were not included in the registry.

The insurance terms, eligibility criteria, compensation structure, and injury reporting procedures remained unchanged throughout the entire study period (2019–2025). No modifications were made to coverage policies in connection with the introduction of body checking.

### Injury Types

In the insurance registry, injuries were originally recorded using detailed injury codes specifying the anatomical location and injury subtype. In their original form, each code represented a distinct injury category, resulting in a large number of narrowly defined groups. For the present study, these detailed codes were aggregated into broader injury categories to facilitate meaningful analyses and prevent sparse data within individual categories.

The main categories were Concussions, Face, Dental, Shoulder, Hand, Knee, Foot and “Other.” Each main category comprises several related, detailed insurance codes that reflect specific anatomical structures within the same region. This aggregation strategy was chosen to ensure clinically meaningful group sizes.

### Statistical Analysis

The injury incidences were presented as IR per 1000 player-game hours with 95% confidence intervals (CI). The distributions of injury type before and after the introduction of body checking were tested using Fisher’s exact test. The significance level was set at *p* < 0.05. All statistical analyses were done in R version 4.4.2 (R Core Team, 2024).

### Ethics

This study was conducted in accordance with the ethical principles outlined in the Declaration of Helsinki. Ethical approval for the project was obtained from the Swedish Ethical Review Authority (Approval number 2025-02641-01). The research is based solely on previously collected and pseudonymized registry data obtained from the Swedish Ice Hockey Association and the players’ mandatory licensing insurance company (Gjensidige). No direct contact with the athletes occurred, and no clinical interventions were performed. Only variables necessary to address the scientific aims of the study were processed. Because this study involves registry-based research using secondary data, informed consent was not required under applicable ethical review legislation.

## Results

In total, 120 injuries were registered among 92 players in the SDHL, which consists of 10 teams with approximately 20–25 players per team (corresponding to around 220 players per season). Descriptive statistics of the study population are presented in Table 1.

**Table 1:** Descriptive characteristics of injured players in the Swedish Women’s Elite Ice Hockey League (SDHL) who filed an insurance claim during the study period (2019–2025)

Characteristics	By injury, *n* = 120	By player, *n* = 92
Age (years), median (Q1–Q3)	20.5 (17–25)	–
Position, *n* (%)		
Forwards	76 (63)	58 (63)
Defensemen	32 (27)	24 (26)
Goalkeepers	12 (10)	10 (11)
Number of injuries, *n* (%)		
1	–	72 (78)
2	–	15 (16)
3–5	–	5 (5)

**Fig. 1:**
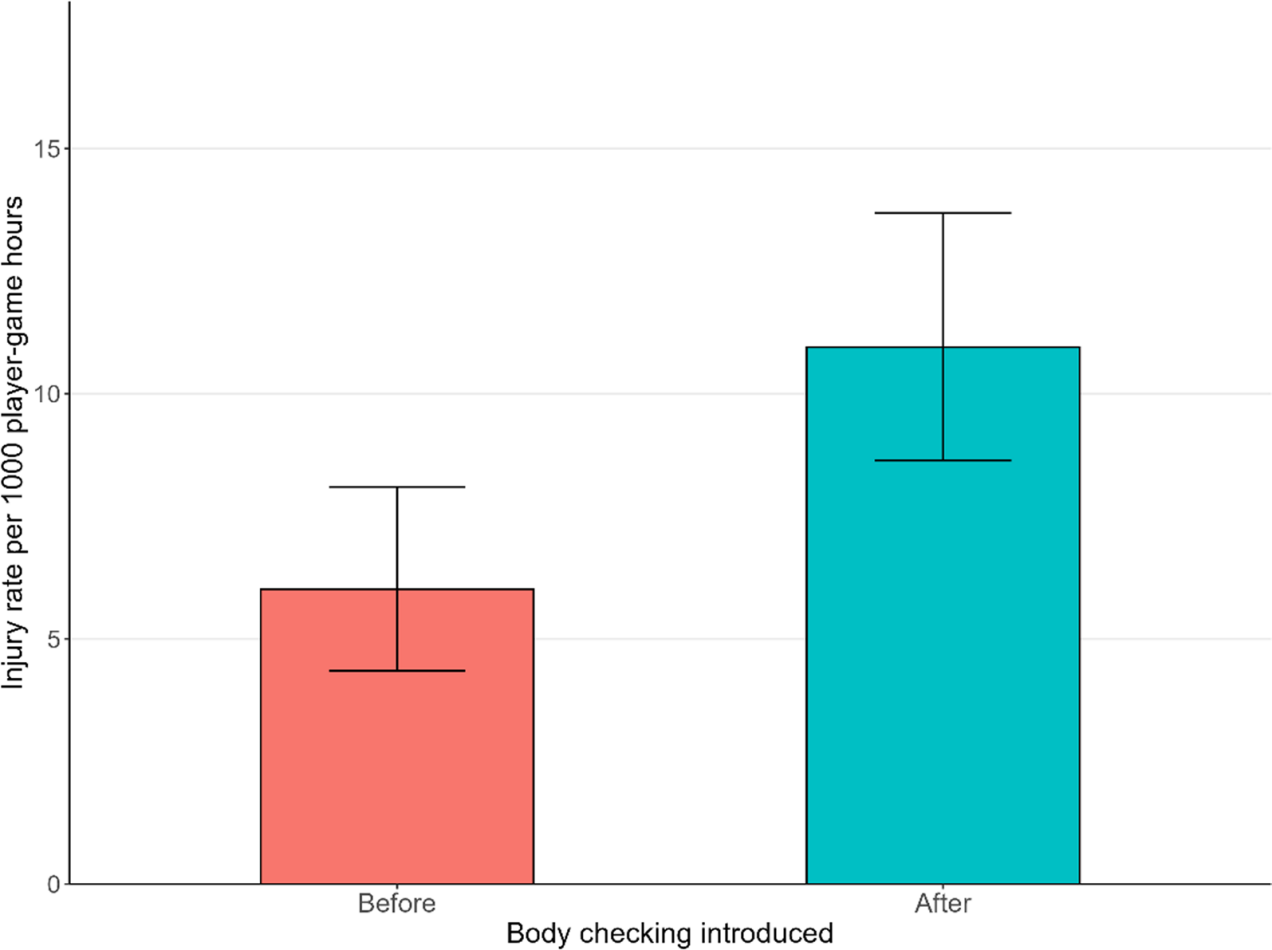
Injury rates per 1000 player-game hours before and after introducing body checking in the Swedish Women’s Hockey League (SDHL) during 2019–2025

Across all seasons, there was a statistically significant increase in the IR during the first season when body checking was introduced (2022–2023; *p* < 0.05), followed by a decline in subsequent seasons (2023–2025), although rates remained elevated compared with the pre-implementation period (2019–2022) (Fig. 1, Table 2).

**Table 2:** Injury rates (IR) per 1,000 player-game hours in the Swedish Women’s Elite Ice Hockey League (SDHL), by season and by pre- and post-implementation periods of body checking (2019–2025)

Season	Number of injuries	IR (95% CI)
2019–2020	10	4.3 (2.0, 7.9)
2020–2021	17	7.2 (4.2, 11.5)
2021–2022	16	6.6 (3.8, 10.7)
2022–2023	35	16.7 (11.6, 23.2)
2023–2024	22	9.0 (5.6, 13.6)
2024–2025	20	8.1 (5.0, 12.4)
Season dichotomized		
2019–2020 to 2021–2022	43	6.0 (4.4, 8.1)
2022–2023 to 2024–2025	77	11.0 (8.6, 13.7)

The distribution of injury types before and after the introduction of body checking is presented in Table 3. No statistically significant differences in injury type distribution were observed between the pre- and post-implementation periods. Descriptively, shoulder injuries accounted for 3 (7.0%) of injuries before implementation and 11 (14.3%) after implementation. Knee injuries accounted for 9 (20.9%) and 15 (19.5%), and face injuries for 2 (4.7%) and 9 (11.7%), respectively.

**Table 3:** Distribution of injury types before and after introducing body checking in the Swedish Women’s Hockey League (SDHL) during 2019–2025

Injury type/location	*n* (%)		*p*-value*	IR (95% CI)	
	Before body checking, *n* = 43	After body checking, *n* = 77		Before body checking, *n* = 43	After body checking, *n* = 77
Concussion	9 (20.9)	10 (13.0)	0.36	1.6 (0.6, 2.4)	1.4 (0.7, 2.6)
Dental	0 (0.0)	2 (2.6)		0.0 (0.0, 0.5)	0.3 (0.0, 1.0)
Face	2 (4.7)	9 (11.7)		0.3 (0.0, 1.0)	1.3 (0.6, 2.4)
Foot	6 (14.0)	6 (7.8)		0.8 (0.3, 1.8)	0.9 (0.3, 1.9)
Hand	10 (23.3)	11 (14.3)		1.4 (0.7, 2.6)	1.6 (0.8, 2.8)
Knee	9 (20.9)	15 (19.5)		1.3 (0.6, 2.4)	2.1 (1.2, 3.5)
Shoulder	3 (7.0)	11 (14.3)		0.4 (0.1, 1.2)	1.6 (0.8, 2.8)
Other	4 (9.3)	13 (16.9)		0.6 (0.2, 1.4)	1.8 (1.0, 3.2)

Data are presented as *n* (%) and injury rates (IR) with 95% confidence intervals (CI)

^*^Fisher’s exact test

## Discussion

This study provides the first league- and nationwide assessment of injury incidence following the introduction of body checking in elite women’s ice hockey. Using nationwide insurance registry data, we observed an increase in injury incidence after the rule change, with the highest rates occurring during the first post-implementation season, followed by a partial decline in subsequent seasons relative to this initial peak, although injury incidence remained higher than during the pre-implementation period.

No statistically significant differences in the distribution of injury types were observed between the pre- and post-implementation periods in this relatively small sample. This lack of difference should be interpreted in light of the data source, as the insurance-based registry captures only injuries leading to claims, which likely contribute to the lower overall injury rates observed compared with studies using broader injury definitions (e.g., time-loss injuries or all medical attention injuries). However, descriptively, face, shoulder, and knee injuries accounted for a larger proportion of injuries following the introduction of body checking. Although these observations do not prove causality and should be interpreted with caution, they may provide useful context for future research on body checking-related injury patterns in women’s ice hockey.

One plausible explanation for the observed increase in injury incidence is that female players have historically developed their sport-specific skills in an environment without intentional body contact. Limited exposure to body checking throughout development may influence neuromuscular preparedness, bracing strategies, and collision technique when full contact is introduced at the elite level [7]. In addition, protective equipment and rule frameworks in ice hockey have largely been developed based on male physiology and playing patterns, which may offer suboptimal protection for female players as collision intensity increases.

One possible explanation for the observed increase in shoulder injuries is that players may be exposed to new contact situations following the introduction of body checking. For example, receiving a check from an outstretched arm may increase the mechanical load on the shoulder joint. It is known from men’s ice hockey that most of shoulder injuries occur from contact with board, which would also explain the increase in shoulder injuries observed in the present study [18]. However, this interpretation remains speculative and requires confirmation in future studies using video analysis or detailed injury surveillance data.

In contrast to several previous studies on body checking in youth and men’s ice hockey, which have reported substantial increases in concussion incidence following the introduction of body checking [8, 19–22], the present study did not observe a significant increase in concussions. One explanation may be insufficient statistical power due to the relatively small size of the study population. However, the discrepancy may also partly be explained by differences in injury definitions and surveillance systems. Unlike injuries such as dental trauma, facial lacerations, or knee injuries, which almost invariably lead to insurance claims due to the need for imaging or specialist care, sport-related concussions may be managed within team medical settings without activating the insurance system. Concussion is a clinical diagnosis, and routine neuroimaging (CT or MRI) is not recommended in uncomplicated cases. As access to imaging is often necessary for initiating insurance claims, this may result in underrepresentation of concussions in insurance-based datasets.

The increased number of facial and dental injuries observed after the introduction of body checking in the present study occurred despite the mandatory use of face grids in women’s ice hockey. Facial injuries also occur in the boys-under-18 category, where players also wear full-face grids [23]. These findings indicate a need for continued evaluation of the protective equipment in ice hockey. Importantly, body checking in women’s ice hockey may not necessarily mirror that in the men’s game, nor should they be assumed to do so. This underscores the need for sex-specific research to better understand the body checking mechanism and appropriate safety strategies specifically for women’s ice hockey.

The decline in injury observed after the initial post-implementation season suggests a potential adaptation effect over time. This pattern may indicate a short-term transitional phase following rule implementation rather than a continuously increasing injury trajectory. It may reflect improvements in player technique, increased on-ice awareness and anticipation of contact situations, or greater familiarity with physical contact. However, these interpretations are speculative and should be interpreted with caution. Future studies incorporating detailed exposure data and biomechanical analyses are needed to determine whether true adaptation effects occur following the introduction of body checking.

Importantly, body checking itself may not be the sole contributing factor to the increased incidence of injuries observed after the rule change. Rather, changes in match dynamics, such as higher game intensity, increased speed, or cumulative fatigue, may also contribute to injury risk. The rule change may also have influenced the frequency and nature of physical engagements, which could further affect injury risk. In addition to body checking, injuries in ice hockey may also result from factors such as high game speed, illegal hits, and unintentional collisions, which could contribute to the observed injury patterns.

Although body checking was associated with higher IRs in this study, the findings should be interpreted within a broader context of sport development and gender equity [3]. While player safety and health remain the primary considerations in ice hockey, physical contact is a core component of the sport, and this policy change may contribute to the perception of women’s ice hockey as a physically demanding and high-intensity sport. Notably, Swedish female elite players have expressed positive attitudes toward the introduction of body checking [2], and preliminary findings from this research project indicate that this attitude persists after 2 years of exposure to the rule change [24, unpublished]. The present study does not take a position on whether body checking should be permitted; rather, it provides evidence that may inform future decision-making. While safety considerations should remain paramount, other factors, including gender equity and players’ readiness and willingness to engage in physical play, may also be relevant when evaluating the introduction of body checking in women’s ice hockey.

In elite women’s ice hockey, an IR of 22.0 injuries per 1,000 player-game hours has been reported during the Women’s World Championships, where body checking is prohibited [15, 25]. In men’s elite ice hockey, substantially higher IRs have been reported, including 88.6 injuries per 1,000 game hours in the Swiss professional league, 74.1 in Swedish male elite players, 52.1 during the Olympic Games [14], 47.4 in the Finnish top league and 49.4 in the NHL [11]. The IR discrepancy may stem from differences in skating speed and protective equipment, as players in men’s ice hockey typically wear visors, whereas full-face protection is mandatory in women’s ice hockey. However, the value of such comparisons is limited by methodological differences.

The discrepancies in IRs may for example be explained by differences in injury definitions and surveillance methods, as the present study captured only injuries resulting in insurance claims, whereas most previous studies included time-loss injuries or even all injuries requiring medical attention. In addition, structural and contextual differences between women’s and men’s ice hockey may further contribute to the differences observed in IRs. Men’s ice hockey is characterized by higher skating speeds, the allowance of fighting at certain levels, and differences in protective equipment, such as the use of visors rather than full-face grids all of which may influence IR [6]. Consequently, direct comparisons of IRs across studies and between sexes should be interpreted with caution. On the other hand, studying temporal trends within the same setting and a comparable population, as in the present study, provides complementary relevant information.

Similar to the present study, changes in injury risk have been observed in men’s ice hockey following policy changes both introducing and disallowing body checking [8, 19–22]. This study highlights the need for further research to gain more insight into the impact of body checking on the risk of injury, which will be necessary in the future to support this rule change.

### Strengths and Limitations

A major strength of this study is the long observation period, encompassing multiple seasons both before and after the introduction of body checking, with consistent data collection procedures and injury definitions applied throughout the study period. The use of a single, nationwide insurance registry including all licensed SDHL players minimizes selection bias and ensures comprehensive coverage of medically treated injuries within the league. The natural experiment created by the rule change enables meaningful temporal comparisons within a stable competitive context. Importantly, this study addresses a notable research gap in female athlete health research, by generating novel data on injuries associated with body checking in women’s ice hockey, an area that has not previously been examined.

Several limitations should also be acknowledged. The insurance registry captured only injuries that resulted in contact with the insurance provider and required medical care, meaning that less severe injuries and illnesses that may still have caused time loss were not included. As a result, the reported injury incidence likely reflects more severe injuries and may underestimate the overall injury burden. Illness-related absences were not covered by the insurance system and were therefore not captured. However, even though insurance-based registries do not capture all injuries, similar approaches have been used in previous studies in athletes, supporting their value for identifying injury patterns in athlete populations [13]. Thus, the currently reported injury rates should not be interpreted as reflecting the total injury burden, but rather as indicators of temporal trends for comparisons within-athlete populations over time.

An important limitation is the inability to distinguish between contact- and non-contact injuries, which limits the ability to directly attribute injuries to body checking. Injury classification was performed by insurance personnel without formal medical training, which may have introduced some degree of misclassification. It may be challenging for non-medically trained personnel to distinguish between related diagnostic entities such as head injury, skull injury, and concussion. As a result, some degree of overlap or misclassification between these categories cannot be excluded. Similar challenges may apply to other injury classifications within the registry, as diagnoses were based on insurance documentation rather than standardized clinical assessments performed by sports medicine professionals. Consequently, the injury categorization should be interpreted with this in mind. Additionally, some players may have sought care through private insurance or alternative healthcare pathways, potentially leading to underreporting.

Another limitation relates to the introduction of the insurance system in 2019, which may have been associated with limited awareness during the early seasons. Increased familiarity with the insurance coverage over time could have contributed to higher reporting rates in later seasons, potentially influencing observed time trends. Although reporting awareness may have increased over time, the relatively stable IR observed during the three pre-implementation seasons suggests that the abrupt increase observed in 2022–2023 is unlikely to be explained solely by gradual improvements in insurance familiarity.

As with all observational studies, causality cannot be established; however, the close temporal proximity between the introduction of body checking and the observed increase in injury incidence supports a meaningful association. In addition, it would have been desirable to have access to more detailed information on the injury mechanism, type and severity, but our analyses were limited by data that were accessible from the insurance company. The inability to separate match-related and practice-related injuries represents an additional limitation. The use of game hours as the denominator in the injury rates, while including injuries from both games and practices, represents a methodological limitation. However, similar approaches have been used in previous ice hockey studies, and methodological challenges in accurately quantifying exposure time in team sports are well recognized [11, 12]. An additional consideration is the variation in the number of games between seasons. Such variation may influence player workload and recovery patterns, as seasons with fewer games may allow more rest between matches, potentially affecting injury risk.

Importantly, because the same methodological approach was applied consistently across all seasons, comparisons between pre- and post-implementation periods should remain internally valid. Therefore, the reported injury rates should primarily be interpreted as relative measures within this specific context rather than absolute injury incidence estimates. However, because match injuries are known to occur at substantially higher rates than training injuries in elite ice hockey, and because this limitation applied equally across all seasons, the internal comparison between periods is unlikely to be substantially biased. Finally, comparisons of IRs across studies should be made with caution due to differences in injury definitions, exposure calculations, and injury surveillance methods used in ice hockey research.

## Conclusions

The introduction of body checking in the Swedish Women’s Elite Ice Hockey League was associated with an increased injury incidence, particularly during the first season following implementation. Although no statistically significant differences in injury type distribution were observed, descriptive patterns indicated a larger proportion of face, shoulder, and knee injuries after the rule change. The partial decline in injury incidence in subsequent seasons may suggest a degree of adaptation over time.

Although the observed increase in injury incidence is clinically relevant, these findings should be interpreted with caution given the methodological constraints of the study. Based on this study alone, it is not possible to draw conclusions regarding whether body checking should be permitted or restricted in women’s ice hockey. Rather, the findings highlight the importance of carefully considering how such rule changes should be introduced. Future studies are needed to evaluate strategies for implementing body checking in a way that minimizes the risk of injury.

## Data Availability

The datasets used and/or analyzed during the current study are available from the corresponding author on reasonable request.
